# Effects of Nasal Solution Incorporating Resveratrol and Carboxymethyl-Β-Glucan in Preschool Non-Atopic Children with Wheezing

**DOI:** 10.3390/nu16142197

**Published:** 2024-07-10

**Authors:** Cristiana Indolfi, Costanza Mignini, Francesco Valitutti, Ilaria Bizzarri, Giulio Dinardo, Angela Klain, Michele Miraglia del Giudice, Giuseppe Di Cara

**Affiliations:** 1 Department of Woman, Child and of General and Specialized Surgery, University of Campania Luigi Vanvitelli, 80138 Naples, Italy; cristianaind@hotmail.com (C.I.); michele.miragliadelgiudice@unicampania.it (M.M.d.G.); 2 Department of Pediatrics, University of Perugia, Giorgio Menghini Square, 06129 Perugia, Italy; costanza_mignini@libero.it (C.M.); francesco.valitutti@gmail.com (F.V.); ilaria.bizzarri@unipg.it (I.B.); giuseppe.dicara@unipg.it (G.D.C.)

**Keywords:** resveratrol, wheezing, preschool wheezing, children, non-atopic children, pediatric populations, respiratory infections, nutraceutical, nutraceutics, respiratory tract diseases

## Abstract

Upper respiratory tract infections (URTI) account for more than 80% of wheezing episodes in children with a high incidence of hospitalization in preschool age. Most children with symptoms of wheezing during an URTI are usually non-atopic. As the majority of wheezing episodes resulting from URTI are attributed to viral triggers, several studies have suggested the potential anti-inflammatory and antiviral properties of resveratrol. This study aims to identify the effect of resveratrol for pediatric non-atopic patients with recurrent wheezing triggered by URTIs. We conducted a prospective single-blind study to assess the effectiveness of a short course of nasal solutions incorporating resveratrol and carboxymethyl-β-glucan, administered for 7 days at the onset of URTIs, compared to standard nasal lavage with 0.9% saline solution. A total of 19 patients entered the active group, 20 patients were assigned to the placebo group. The comparison of overall wheezing days (*p* < 0.001), mean wheezing days per month (*p* < 0.01), and wheezing episodes per patient (*p* < 0.001) in the two groups showed a significant reduction in the group receiving resveratrol compared with the placebo group, with less hospital access (*p* < 0.001) and oral corticosteroid administration (*p* < 0.01). Our findings seem to suggest that, in non-atopic children with recurrent wheezing secondary to URTIs, nasal resveratrol could be effective to prevent or reduce the occurrence of wheezing, when started from the onset of upper airway symptoms.

## 1. Introduction

Recurrent wheezing is common among preschool children with various clinical phenotypes and can affect up to half of children under the age of 5. It is a leading cause of visits to the emergency department or referrals to pediatricians [1,2,3]. As explained in the article by Salehian et al., two symptom-based diagnostic presentations can be identified in children with preschool wheeze: episodic viral wheeze (EVW), where symptoms are associated with viral infections, and multiple trigger wheeze (MTW), where symptoms are triggered by various factors, and illnesses and infections can worsen respiratory symptoms [4]. Children with EVW typically stop wheezing by school age, whereas those with MTW continue to have symptoms throughout childhood and are more likely to be diagnosed with asthma later on. However, this classification is not always reliable, as children can transition from one phenotype to another over time [4]. Upper respiratory tract infections (URTIs) represents the main cause in approximately 80% of the episodes of wheezing in childhood [5,6] with high incidence of hospitalization at the preschool age [7,8]. The majority of children with EVW with symptoms by 6 years of age are usually non-atopic [9,10,11]. In this group of patients, the optimal preventive management and treatment remain elusive [12,13]. According to The Individualized Therapy for Asthma in Toddlers (INFANT) trial, children with elevated blood eosinophils and sensitization to inhalant allergens show a beneficial response to daily inhaled corticosteroids therapy differently; in non-atopic children, the efficacy of inhaled therapy is less evident [14]. Looking for the optimal treatment, the role of an intermittent therapy with oral corticosteroid (OCS) has been evaluated in several studies without conclusive results. A short-term course of oral corticosteroids after the onset of viral-induced wheezing seems to modestly reduce the severity and duration of each episode, with no influence on hospitalization rates [15]. More recently, a randomized trial comparing OCS and placebo in a group of 700 children did not show significant differences in terms of severity and duration of hospitalization [16]. Frequent URTIs rank among the primary reasons for pediatric medical consultations during the early years of childhood [2]. Reducing episodes of URTIs could help decrease instances of preschool wheezing. The standard treatment protocols for URTIs typically involve antipyretics, antibiotics, anti-inflammatory drugs, and corticosteroids [2,17]. Despite their effectiveness, these medications often lead to higher healthcare costs and potential adverse effects on young patients. A less invasive strategy for preventing or reducing the occurrence of viral-induced wheezing in children with frequent URTIs is to control nasal inflammation. This approach can reduce acute infectious rhinitis and other inflammatory episodes that trigger bronchoconstriction [18]. The interconnected relationship between the upper airways (nose, sinuses) and the lower airways (bronchi and lungs) is well documented, and it is also named as United Airway Disease (UAD) [19,20,21]. This concept suggests that pathologies affecting the upper and lower airways may be interrelated and mutually influence each other. The upper and lower airways are anatomically and physiologically connected, allowing inflammation or infection originating in one area to spread or trigger a systemic inflammatory response affecting both regions [22]. Conditions such as allergic rhinitis, sinusitis, asthma, and bronchitis frequently coexist in patients, illustrating the overlap in these respiratory disorders. Patients with UAD may experience additional symptoms due to inflammation spreading between these areas, leading to persistent cough, breathing difficulties, and a decline in quality of life. According to this concept, addressing nasal conditions can be effective in managing and preventing lung diseases, and therefore, managing nasal health plays a critical role in maintaining respiratory wellness and overall quality of life [23,24]. The nasal microbiome plays a significant role in maintaining respiratory health, impacting conditions in the lungs as well. Research highlights that the composition and balance of microorganisms in the nasal cavity can influence the lower airways. This interconnected relationship occurs through the aspiration of nasal secretions into the lungs, affecting respiratory conditions such as asthma, chronic obstructive pulmonary disease (COPD), and respiratory infections [25,26]. A diverse and stable nasal microbiome contributes to a healthy immune response and serves as a barrier against pathogens. Conversely, disruptions or dysbiosis in the nasal microbiome have been linked to increased susceptibility to respiratory diseases and poorer treatment outcomes [27,28,29]. Understanding this connection opens avenues for therapeutic strategies aimed at restoring or maintaining a healthy nasal microbiome, potentially benefiting overall respiratory health. In recent years, many studies have highlighted several molecules with promising activity in reducing the frequency and severity of upper airway infections. However, few studies focused on the impact of improved control on lower airway responses [30,31]. Consequently, modern research is shifting towards investigating alternative treatments and preventive strategies, particularly those involving nutraceuticals [32,33,34]. In the nutraceuticals family, the promising molecule resveratrol has garnered significant interest due to its potential health benefits and has been approved by the European Food Safety Authority (EFSA) for use as a novel food supplement [32,35]. Resveratrol is a polyphenolic antioxidant commonly found in nature, part of the stilbene family, characterized by a distinctive carbon skeleton of C6–C2–C6 (1,2-diphenylethylene). Resveratrol is commonly present in fermented grapes, mulberry, red wine, and peanuts. It functions as a phytoalexin (a class of vegetal antibiotics) protecting the plant from environmental stress or infections [36]. Several studies have investigated biological effects of resveratrol, suggesting a role in inhibiting cancer progression [37], a protective role on microangiopathy and coronaropathy onset [38,39,40,41] and as a regulator in inflammatory diseases and viral infections [31,42,43]. The anti-inflammatory actions of resveratrol are largely attributed to its inhibition of the NF-kB transcription factor, particularly by targeting Ik-B kinase [44]. In mouse models of allergic asthma, resveratrol has exhibited anti-inflammatory and anti-asthmatic benefits by lowering IL-4 and IL-5 concentrations in plasma and bronchoalveolar lavage fluid, as well as reducing bronchial hyperreactivity, lung eosinophilia, and mucus overproduction [45]. However, resveratrol exhibits low bioavailability, resulting in inefficient absorption and utilization by the body when ingested via diet or supplements [46]. In order to increase the bioavailability of resveratrol, several studies have recommended an aqueous liquid formulation that incorporates resveratrol with a modified glucan known as carboxymethylated (1,3/1,6)-β-D-glucan [47,48]. β-glucans are polysaccharides made up of β-glucose units, characterized by glycosidic bonds predominantly at β(1,3) and additional linkages at β(1,6). They are derived from various sources including fungi, cereals such as oats and barley, bacteria, and seaweeds. These natural polysaccharides are known as ‘biological response modifiers’. They activate the immune system, offering benefits such as anticancer and antiviral effects. When 1,3-β-glucans interact with immune cells, they trigger powerful immunomodulating activities. This includes the stimulation of cytokine production, increased proliferation of phagocytes and lymphocytes, an oxidative burst, and enhanced antioxidant response [49]. In pre-clinical research conducted by Francioso et al., it was discovered that the combination of resveratrol plus carboxymethyl-β-glucan solution prevents the degradation of resveratrol in water and effectively combats rhinovirus infection in human nasal epithelial cells by influencing viral replication and the expression of inflammatory mediators. The use of this combination as a nasal spray for treating upper respiratory tract infections was proven to be safe and effective. Furthermore, numerous studies have shown that β-glucan and resveratrol together have a synergistic effect in boosting the immune system and significantly lowering stress-related markers such as corticosterone, IL-6, IL-12, and interferon (IFN)-γ, compared to when these compounds are used individually [50,51]. Moreover, recent clinical trials have examined the potential antiviral benefits of a solution containing resveratrol and carboxymethyl-β-glucan for children with respiratory diseases. These trials demonstrated that the synergistic action of carboxymethyl-β-glucan effectively reduced symptoms and improved quality of life in patients with respiratory infections, as well as in those with allergies and respiratory inflammation [18,31,52]. Based on the evidence about its anti-inflammatory activity and considering the mainly viral trigger in preschool wheezing, our study aimed to prospectively evaluate the efficacy of a short course of nasal solution incorporating resveratrol and carboxymethyl-β-glucan, administered for 7 days at the onset of upper airway symptoms, before the occurrence of wheezing, compared to standard nasal lavage with 0.9% saline solutions.

## 2. Materials and Methods

### 2.1. Patients

Patients who experienced recurrent wheezing in the past 12 months were enrolled from April to August 2022. After a wash-out period in September 2022, they were randomly assigned to two groups using a straightforward randomization process. This study was single-blind and placebo-controlled.

Placebo group: patients treated with 0.9% saline solution daily nasal lavage, once a day, for the whole follow-up period.

Active group: patients treated with 0.9% saline solution daily nasal lavage once a day, for the whole follow-up period and a 7-day add-on therapy with isotonic solutions containing resveratrol 0.05% (extracted by Polygonum cuspidatum) and carboxymethyl-b-glucan 0.33% from each onset of upper airways symptoms. The nasal resveratrol/carboxymethyl-β-glucan solution was produced by Noos srl, Rome, Italy, and currently sold in Italy as a Class I EC Medical Device, under the brand Linfovir^®^ plus.

The inclusion criteria were as follows:

- Presence of at least 8 episodes of wheezing in the previous 6 months;
- Negative skin prick test (SPT) for the most common allergens;
- Age less than 6 years;
- Preschool program attendance both in the previous year and in the follow-up year.

The exclusion criteria were as follows:

- Sensitization to food allergens;
- Sensitization to perennial allergens or tree pollen allergens;
- Presence of atopic dermatitis;
- Background treatment with montelukast and/or inhaled corticosteroids;
- Presence of comorbidities for infectious or respiratory diseases (such as immune disorders, cardiopathy, pulmonary disease, and preterm birth).

After the enrolment, patients were instructed to record symptoms of upper and lower airways during the one-month wash-out period. All patients were evaluated in November 2022 and in March 2023, after six months. All patients were instructed to register in a dedicated chart the number of days with symptoms of upper airway inflammation and the recurrence of wheezing. As a predictive index of severity, parents were instructed to register the number of days with OCS administration. All patients were instructed to treat each wheezing episode, from its onset, with inhaled salbutamol according to weight and, if wheezing persisted, by adding oral prednisolone once a day, according to the guidelines [53]. Patients in the active group were also instructed to treat each upper airway episode with nasal solutions incorporating resveratrol and carboxymethyl-β-glucan 4 times a day for 7 consecutive days. Hospitalizations and ER admissions were documented when they occurred. Written informed consent was acquired from a parent or guardian for all participants. This study has been approved by the Ethical Committee of the Healthcare Companies (CEAS) of Umbria in Italy, approval number 12904/18/ESS.

### 2.2. Skin Prick Tests

The sensitization status of each patient was assessed by performing skin prick tests (SPTs) with a standard panel of environmental (Stallergenes, Antony, France) and food allergens (Lofarma, Milan, Italy). The positivity of SPTs was established according to the guidelines of the European Academy of Allergology and Clinical Immunology [54]. The standard panel of allergens included phleum pratense, parietaria judaica, juniperus ashei, olea europaea, dermatophagoides pteronyssinus, and alternaria tenuis; cat and dog epithelium; milk, egg, peanut, tree nuts, fish, shellfish, soy, and wheat; and a positive (histamine 10 mg/mL) and a negative (normal saline) control. SPTs with wheals measuring ≥ 5 mm were considered positive.

### 2.3. Clinical Symptoms, Rescue Medication, and Severity Evaluation

During the evaluation period, patients filled in daily diary cards to record days with symptoms involving the upper (sneezing, rhinorrhoea, itching, and nasal blockage) and lower airways (cough, dyspnea, and wheezing) and the use of OCS. The clinician recorded days of hospitalization when it occurred.

### 2.4. Statistical Analysis

For database construction and data analysis, all continuous variables were assessed using the Shapiro–Wilk test. The Mann–Whitney U-test was used for intergroup analysis, and the Wilcoxon test for paired data was used for intragroup analysis. Medians with interquartile ranges (IQRs) are presented for these non-normally distributed data. Values of *p* lower than 0.05 were considered statistically significant. All statistical analyses were made using the standard statistical software SPSS 25.0.

## 3. Results

A total of 42 children were included in this study. After the wash-out period, 40 patients presented at visit 2 at the end of September and were randomized into the two groups of 20 patients each. At the end of follow-up, one patient in the active group was excluded from this study because he did not complete the follow-up. A total of 39 patients (24 males and 15 females, mean age 4.2 years, range 3.6–5.0 years) completed the follow-up.

### 3.1. Upper Respiratory Infection Episodes

**Figure 1:**
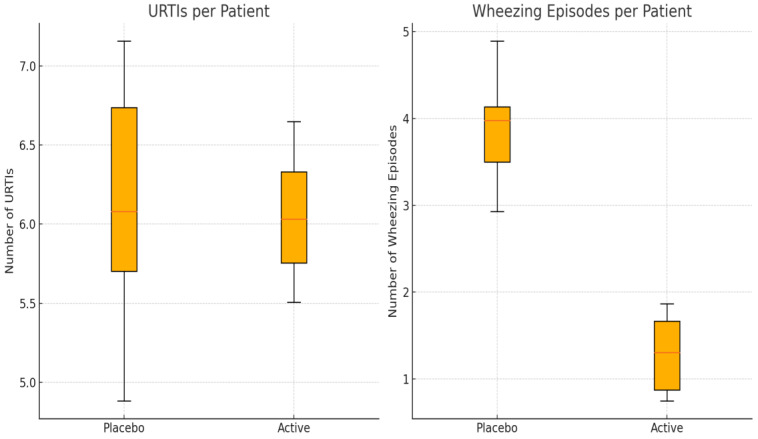
Distribution of URTIs and wheezing episodes in placebo and active groups.

Patients in the placebo group showed a total of 130 episodes of URTI symptoms, with a median (IQR) of 6.03 (2.31) episodes per patient in the follow-up period and a mean of 6.1 days/month with symptoms; in the active group, a total of 117 episodes of URTI symptoms were reported, with a median (IQR) of 6.05 (1.36) episodes per patient in the follow-up period and a mean of 4.9 days/month with symptoms. No significant differences between the two groups in terms of number and median of episodes per patient were reported (Figure 1) (Table 1).

### 3.2. Wheezing Episodes

When comparing total wheezing days in the two groups, the placebo group showed a total of 86 episodes of wheezing, with a median (IQR) of 3.89 (2.05) episodes per patient and 2.1 days/month. Conversely, patients in the active group reported 23 episodes of wheezing, with a median (IQR) of 1.29 (1.29) episodes per patient and 0.5 days/month, demonstrating a significant reduction when compared with the placebo group (Figure 1) (Table 1).

### 3.3. Severity Index

During the follow-up period, the placebo group presented a mean of 0.6 days/month with oral corticosteroid administration or hospitalization. In particular, a total of 18 days were reported with hospital access and 53 days with corticosteroid administration. A significant reduction was present in the active group, with a mean of 0.1 days/month with oral corticosteroid administration or hospitalization and a total of 1 day with hospital access and 10 days with corticosteroid administration (Table 1).

### 3.4. Safety

No serious adverse events (SAE) were reported during the follow-up period. The most common mild adverse effect in active group patients was nasal irritation after administration of solutions incorporating resveratrol and carboxymethyl-β-glucan, which was present for a few minutes and commonly resolved without any therapy. Such symptoms were reported in 15/20 patients in the active group and in 8/20 patients in the placebo group. However, none of the patients in the active group needed to discontinue the treatment.

## 4. Discussion

Our findings seem to suggest that, in non-atopic children with recurrent wheezing secondary to URTIs, the use of nasal solutions incorporating resveratrol and carboxymethyl-β-glucan could be effective in preventing and/or reducing the occurrence of wheezing; an intermittent schedule of administration could also be effective, when started from the onset of upper airway symptoms. Our patients exhibited marked enhancements in both the frequency and severity of wheezing episodes compared to those who received the placebo, with no notable variance in adverse effects. A similar occurrence of upper airway infections in both groups is not unexpected, as resveratrol was not used as a therapy for preventive purposes in our study. Indeed, considering its primary antiviral action targeting the early stages of viral replication, initiating resveratrol treatment within the first hours of infection may be of help in observing differences in the frequency and duration of upper airway episodes among patients. The protective role of resveratrol in several respiratory diseases and its possible importance in contrasting the oxidative stress secondary to exposure to smoking and pollution has been suggested in clinical studies which highlighted its activity in inhibiting the production of reactive oxygen species (ROS) by neutrophils [55] and in reducing the impact of free radicals through the production of specific endogenous antioxidant molecules [56]. About its specific activity against viral infections, resveratrol showed an in vitro inhibiting activity against RSV replication and a reduction in its inflammatory effects, in terms of cytokine production and toll-like receptor expression [57]. A similar antiviral effect has been described also against the influenza virus, Epstein–Barr virus (EBV), Herpes Simplex (HSV), Varicella–Zoster virus (VVZ) and Cytomegalovirus (CMV) [58]. Rossi et al. also reported significant antiviral activity against SARS-CoV-2, suggesting a potential role as an adjunctive antiviral agent, through the inhibition of viral replication in human primary bronchial epithelial cell cultures [59]. Moreover, considering the leading role in asthma and wheezing exacerbations, an important activity of resveratrol was also evidenced in inhibiting the replication of human rhinovirus (HRV), with reduced expression of inflammatory mediators in upper airways [60]. Furthermore, resveratrol has gained significant attention in the scientific community for its antibacterial proprieties [61]. Bacteria frequently use biofilm formation as a means of survival, building communities that are resistant to antimicrobial treatments and external pressures. In pre-clinical studies, resveratrol has shown notable antibiofilm activity against both Gram-positive and Gram-negative bacteria, moreover [62].

### 4.1. Resveratrol plus Carboxymethyl-β-Glucan in Pediatric Respiratory Trials

The clinical efficacy of nasal solution of resveratrol plus carboxymethyl-β-glucan towards URTI has also been suggested in real-life studies. A case-control trial by Varricchio et al., involving nasal resveratrol plus carboxymethyl-β-glucan in patients aged 6–11 years with recurrent URTIs, demonstrated significant improvements in both clinical outcomes and quality of life. In this study, 82 children with acute rhinopharyngitis and RRIs were treated with either resveratrol plus carboxymethyl-β-glucan or an isotonic saline solution for 20 days, following a 10-day anti-infective and anti-inflammatory regimen. Evaluations conducted up to 90 days post-treatment showed that the active compound significantly reduced symptoms such as nasal obstruction, rhinorrhea, sneezing, cough, and fever, as well as the need for medication, medical visits, and school absences [52]. A preliminary study conducted by Miraglia del Giudice et al. reported encouraging outcomes in alleviating nasal inflammation, as seen in other inflammatory conditions that could elevate Intercellular Adhesion Molecule-1 (ICAM-1) expression and susceptibility to viral infections. In this study, nasal administration of a combination of resveratrol and carboxymethyl-β-glucan resulted in reduced symptoms and medication usage among children diagnosed with allergic rhinitis [31]. Moreover, in a study by Baldassarre et al., it was observed that a nasal resveratrol/carboxymethyl-β-glucan solution showed potential benefits for infants with respiratory infections compared to a saline solution. The study involved 89 infants and assessed improvements in common cold symptoms using the CARIFS score, the presence of rhinovirus and cytokine expression, and the frequency of relapses over 30 days. The results indicated that while both groups showed improvement in CARIFS scores, the resveratrol/β-glucan group experienced fewer sneezing and coughing episodes after 7 days (*p* < 0.05) [63]. A recent study by Tosca et al. aimed to assess the efficacy and safety of a novel nasal spray containing lactoferrin, dipotassium glycyrrhizinate, carboxymethyl-beta-glucan, and vitamins C and D3. The study included preschool children with frequent respiratory infections and wheezing. Participants were divided into two groups: one received the nasal spray as an add-on to standard treatment, while the control group received only standard treatment. The primary outcomes measured were the number of respiratory infections and wheezing episodes, along with medication use and symptom severity. The results showed that children using the nasal spray experienced significantly fewer respiratory infections and reduced the use of beta-2 agonists compared to the control group (*p* = 0.01 and 0.029, respectively). Unlike our research, this study did not use a spray containing both resveratrol and carboxymethyl-beta-glucan, the nasal spray was used as an add-on therapy, and the population was both allergic and non-allergic [64].

### 4.2. Prospective and Limitations

According to these clinical trials, formulations containing resveratrol and carboxymethyl-β-glucan have shown promising results in treating children with respiratory diseases. Specifically, these studies have demonstrated that these formulations significantly reduce symptoms of respiratory infections and allergic rhinitis. In contrast, based on our experience, nasal formulations containing resveratrol and carboxymethyl glucan have not shown a significant impact on respiratory infections. This discrepancy highlights the need for further research to clarify the effectiveness of resveratrol in respiratory infections. Moreover, it is important to highlight that this nutraceutical is not a pharmaceutical drug and should not be considered a replacement for established treatments. Instead, it should be seen as an adjunct therapy, meant to complement standard treatments rather than replace them [46]. In all those studies, resveratrol was used with an intermittent treatment regimen, during upper airway symptoms. More data would be required to define the treatment regimen and its possible role in preventing further upper airway infections when used continuously. Another limitation is that our study did not include specific data collection or analysis regarding bacterial superinfections or the need for antibiotics use during the study period. Such information would provide valuable insights into the wider effects of resveratrol on the incidence and progression of URTIs in children. However, the need for antibiotics in URTIs is minimal, with only a few cases requiring them and our study aimed to focus on wheezing episodes triggered by URTIs rather than URTIs requiring antibiotic therapy. To the best of our knowledge, no similar data have been previously presented on the impact of resveratrol in lower airway symptoms. However, to confirm this evidence, we require additional data and a larger patient cohort, including diverse populations. Expanding the study to include a greater number of participants from different demographic backgrounds would strengthen the reliability and generalizability of the findings. The results evidenced in our study could be explained by the peculiar phenotypic characteristics of this group of patients. Indeed, all our children had no allergen sensitization, and this feature is more often linked to viral-trigger wheezing without persisting lung inflammation between each episode. The efficacy of this treatment in atopic children, with allergic or multi-trigger wheezing, should be tested to verify or exclude possible different results. The role of a persisting inflammation, such as in allergic and especially in polysensitized patients, could impact and interfere with lower airway symptoms development. At the moment, to our knowledge, there are only a few studies evaluating resveratrol’s clinical effect in allergic patients with allergic disease, in which its additional administration improved upper airway symptoms, such as nasal obstruction, itching, sneezing, and rhinorrhoea, but no data about lower airways are reported [65].

## 5. Conclusions

Our data suggest that intermittent therapy with nasal solutions incorporating resveratrol and carboxymethyl-β-glucan could be effective in reducing the occurrence of wheezing in non-atopic preschool children. This therapy should be considered in patients with a previous history of frequent lower respiratory symptoms. Based on these data, this treatment option could also be considered in non-atopic children who have presented significant side effects during other preventive treatments.

## Figures and Tables

**Table 1:** Comparison of Active Treatment and Placebo Effects.

Group	1 (Placebo)	2 (Active)	*p*
n. patients (male)	20 (12)	19 (12)	
Upper respiratory infections			
Total n.	130	117	NS ^1^
Episodes/patient (median [IQR])	6.03 [2.31]	6.05 [1.36]	NS ^1^
Days/month (mean)	6.1	4.9	NS ^1^
Wheezing episodes			
Total n.	86	23	<0.001
Episodes/patient (median [IQR])	3.89 [2.05]	1.29 [1.29]	<0.001
Days/month (mean)	2.1	0.5	<0.01
Severity index			
Total n. hospitalization (days)	18	1	<0.001
Total OCS ^1^ administration (days)	53	10	<0.01
Severity days/month (mean)	0.6	0.1	<0.01

^1^ List of abbreviations: OCS, oral corticosteroids; NS, not significative.

## Data Availability

Data are available from the corresponding author upon reasonable request.
